# Natural killer cells are crucial for the efficacy of Icon (factor VII/human IgG1 Fc) immunotherapy in human tongue cancer

**DOI:** 10.1186/1471-2172-11-49

**Published:** 2010-10-12

**Authors:** Zhiwei Hu, Jing Li

**Affiliations:** 1 Department of Obstetrics, Gynecology and Reproductive Sciences, Yale University, New Haven, CT 06520, USA; 2 Department of Molecular Biophysics and Biochemistry, Yale University, New Haven, CT 06520, USA

## Abstract

**Background:**

Icon is a novel, dual neovascular- and cancer cell-targeting immunotherapeutic agent and has shown efficacy in the treatment of cancer, wet form macular degeneration and endometriosis. However, its underlying mechanism remains to be investigated. The objective of this study is to elucidate the mechanism of Icon immunotherapy in cancer using a squamous carcinoma human tongue cancer line TCA8113 *in vitro *and *in vivo *in severe combined immunodeficiency (SCID) mice.

**Results:**

We showed that Icon, as a chimeric factor VII and human IgG1 Fc immunoconjugate, could separately induce murine natural killer (NK) cells and activate complement to kill TCA8113 cancer cells *in vitro *via antibody dependent cell-mediated cytotoxicity (ADCC) and complement-dependent cytotoxicity (CDC). However, Icon-NK ADCC had a significantly stronger effect than that of Icon-CDC. Moreover, Icon could completely eradicate established human tongue tumour xenografts *in vivo *in the CB-17 strain of SCID mice that have functional NK cells at a normal level, whereas it was less effective in SCID/Beige mice that do not have functional NK cells.

**Conclusions:**

We conclude that NK cells are crucial for the efficacy of Icon immunotherapy in the treatment of cancer. The results also suggest that impaired NK level/activity could contribute to the resistance to therapeutic antibodies that are currently under investigation in preclinical and clinical studies.

## Background

Several prevalent diseases such as cancer, exudative (wet) age-related macular degeneration, diabetic retinopathy, and rheumatoid arthritis are associated with abnormal angiogenesis, i.e., formation of pathological neovasculature. It is believed that targeting pathological neovasculature is a better strategy for cancer therapy than targeting tumour cells [1]. In the case of cancer, there are two methods for targeting pathological neovasculature in tumours, namely anti-angiogenesis by anti-angiogenic inhibitors [2] and anti-neovasculature by vascular disrupting agents [3-5]. Because pathological neovasculature usually has formed by the time a diagnosis is reached, eradication of the pathological neovasculature is necessary to achieve optimal therapeutic efficacy. Among those vascular disrupting agents there are several molecules called vascular targeting agents [6]. These vascular targeting agents were designed to bring soluble tissue factor (TF) to tumour endothelial cells by targeting MHC class II, cell adhesion molecules, fibronectin, or prostate specific membrane antigen, and then to cause the shutdown of the blood vessels of tumours by initiating blood clotting. The vascular targeting agents are expected to show the greatest therapeutic benefit for anti-neovasculature treatment as part of combined modality regimens [7].

The anti-pathological neovasculature protocol that Garen and Hu developed [8-10] is different from anti-angiogenic inhibitors and those TF-containing vascular targeting agents. Our protocol is based on a chimeric antibody-like molecule, named Icon, composed of two mature coagulation factor VII (fVII) peptides, the natural ligand for receptor tissue factor (TF), fused to the Fc domain of a human IgG1 antibody by recombinant DNA technology. TF, a normal cell surface receptor [11,12], is expressed on tumour vascular endothelial cells [13,14], which can be induced by tumour cell-produced VEGF and other growth factors [15,16], but not on normal vascular endothelial cells [17-20]. Although TF is expressed on extravascular cells of several normal tissues and in the adventitial layer of the blood vessel wall, it is sequestered by fVII at these sites by the tight endothelial cell layer of the normal vasculature [17-19]. In addition, TF is over-expressed on many types of solid cancer cells [8-10,13,14]. Thus, TF provides a common but specific target in tumour neovasculature and tumour cells for development of novel cancer therapies and diagnostic protocols.

The Icon protein can specifically target both tumour cells and tumour vascular endothelial cells via binding TF for the treatment and diagnosis of cancer. The Icon molecule is designed to bind to TF with far higher affinity and specificity than can be achieved with an anti-TF antibody. Icon has several important advantages as compared to an anti-TF monoclonal antibody. The Kd for fVII binding to TF is up to 10^-12 ^M [21], in contrast to anti-TF antibodies that have a Kd in the range of 10^-8 ^to 10^-9 ^M for TF [22]. Icon is produced by recombinant DNA technology, allowing mouse Icon (mouse fVII/human IgG1 Fc) to be made and tested in animal models of diseases and human Icon (human fVII/human IgG1 Fc) to be made from human sources for future clinical trials without the need of the humanisation process that is required for monoclonal antibodies. Tests of Icon immunotherapy in mouse models of primary and metastatic tumours have demonstrated that primary and distant metastatic tumours can be eradicated without causing obvious toxicity [9,10,23]. In addition, Icon was efficacious and safe for the eradication of choroidal neovascularisation in mouse and pig models simulating wet-form macular degeneration [24,25] and for eradication of the neovasculature in human endometriosis in a mouse model [26].

Recently we showed that peripheral blood lymphocytes could induce Icon-dependent cytotoxicity to human endometrial cancer cells *in vitro *[27]. However, the underlying killing mechanisms of Icon immunotherapy remain to be further defined. The objective of this study is to define these mechanisms of Icon immunotherapy for cancer *in vitro *and *in vivo *using a squamous carcinoma human tongue cancer line TCA8113 [28]. Because Icon is an antibody-like molecule, presumably natural killer (NK) cells and complement should be separately involved in the killing actions through antibody-dependent cellular cytotoxicity (ADCC) and complement dependent cytotoxicity (CDC) pathways. NK cells are highly specialised lymphoid cells involved in ADCC against tumour or virally infected cells. Here, we test Icon-mediated ADCC and CDC effects on human tongue cancer *in vitro *and *in vivo *in two genetically modified SCID mouse strains [29], namely SCID/CB-17 and SCID/Beige. The SCID/CB-17 strain has functional NK cells but lack mature B- and T-lymphocytes [30], whereas the SCID/Beige strain not only lacks B-and T-lymphocytes but also has defective NK cells [31,32]. Therefore, NK cell-mediated ADCC is only active in the CB-17 strain, whereas both mouse strains have complement proteins that could mediate CDC. This paper reports for the first time that Icon as an antibody-like immunoconjugate containing human IgG1 Fc can initiate NK cell-mediated ADCC for killing human cancer cells *in vitro *and that NK cell level/activity is crucial for the efficacy of Icon immunotherapy of human cancer *in vitro *and *in vivo*.

## Results

### The mouse Icon protein can be produced by Icon producer CHO cells and adenoviral vector-infected tumour cells and can bind to human tongue cancer cells

**Figure 1:**
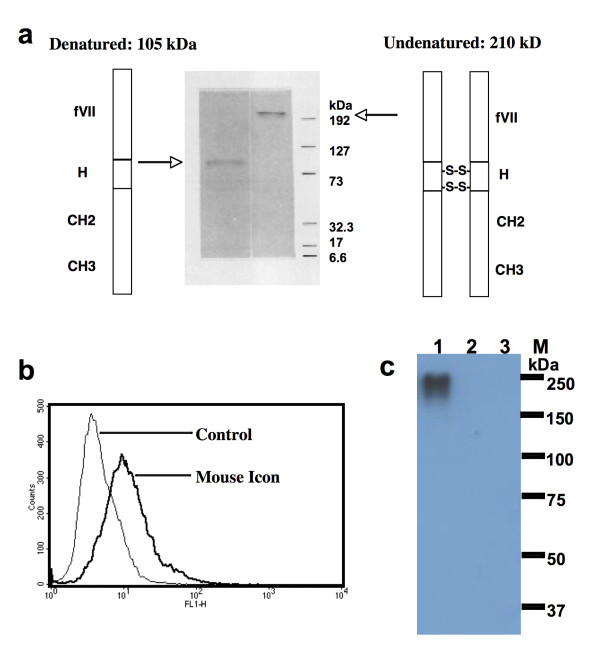
**Production and binding activity of Icon protein**. **a**. Molecular weight of the Icon protein produced by CHO cells analysed by SDS-PAGE. fVII: mouse factor VII with K341A mutation; H: hinge region of a human IgG1 Fc; CH2 and CH3: the second and third domains of the constant region on the heavy chain of a human IgG1 Fc. **b**. Binding activity of Icon protein to human tongue cancer TCA8113 cells by flow cytometry. Control: TCA8113 cancer cells were not incubated with Icon but with secondary antibody FITC. Mouse Icon: the cells were incubated with Icon protein then with the secondary antibody to human IgG Fc FITC. **c**. Immunoprecipitation Western-blotting analysis of Icon protein production by TCA8113 cells one day after infection with AdmIcon (lane 1) or AdBlank (lane 2). The serum free culture medium from uninfected TCA8113 cells was used as uninfected control (lane 3). M: Protein markers (Bio-Rad All blue). Molecular weights (kDa) of the protein markers are indicated. Data are representative of two experiments.

As shown in SDS-PAGE (Figure 1a), the molecular weight of mouse Icon (mfVII/hIgG1 Fc, GenBank accession no AF272773) produced by Icon producer CHO cells was 210 kDa under non-reducing conditions, and 105 kDa under reducing conditions with β-mercaptoethanol. These results suggested that Icon is a homodimeric molecule composed of two identical single peptides, each with a molecular weight of 105 kDa. Flow cytometry analysis showed that the Icon protein could bind to human tongue cancer cells (Figure 1b).

Icon was also detected by Western-blotting in a serum-free culture medium of human tongue cancer TCA8113 cells after infection with an adenoviral vector encoding GFP and Icon (AdmIcon), but not in the medium of the cells infected with a control vector AdBlank (Figure 1c). This result provides a link for understanding the *in vitro *efficacy using Icon protein and the *in vivo *efficacy using Icon vector, as reported below.

### NK cells mediate Icon-ADCC on human tongue cancer cells

**Figure 2:**
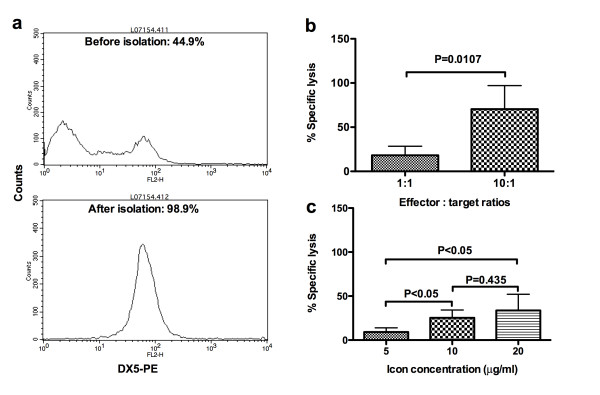
**Lysis of TCA8113 cancer cells by Icon-dependent NK cell-mediated ADCC and complement-mediated CDC *in vitro***. **a**. Isolation of murine NK cells from a single cell suspension of splenocytes from SCID/CB-17 mice. **b**. Icon-dependent NK cell-mediated ADCC. **c**. Icon-dependent complement-mediated cytotoxicity. Note that % specific lysis was derived from the % of cytotoxicity of Icon + NK cell or Icon + complement subtracted by % of cytotoxicity of NK cell alone or complement alone. P values and no significance (ns) were analysed by unpaired t test (b) or one way ANOVA with Tukey's Multiple Comparison Test (c). Data are representative of two experiments.

The NK cells used in ADCC assays were isolated from murine splenocytes of SCID/CB-17 mice using a rat monoclonal anti-mouse NK cell DX5 antibody. DX5 antibody can bind to mouse NK cells without strain limitation [33]. Before isolation, pan NK positive cells were 44.9% in splenocytes using a DX5-PE conjugate as determined by flow cytometry (Figure 2a). After isolation, the NK percentage increased to 98.9% (Figure 2a). These purified NK cells were used for Icon-ADCC below.

Using the Calcein-AM dye release assay, murine splenic NK cells in the presence of mouse Icon protein (10 μg/ml) killed 18% and 70% of human tongue cancer cells at effector:target (E:T) ratios of 1:1 and 10:1 respectively (Figure 2b) (two-tailed P = 0.0107 by unpaired t test). Note that the direct effect of NK cytotoxicity in the absence of Icon protein was subtracted from these percentages of specific lysis. Thus, the results suggested that mouse NK cells could mediate mouse Icon, which contains a human IgG1 Fc as effector domain, for specific lysis of cancer cells through ADCC mechanism.

### Icon-CDC is less effective at killing human tongue cancer cells than Icon-ADCC mediated by NK cells

We showed that the Icon-mediated CDC effect was dependent upon the concentration of Icon protein (two-tailed P < 0.05 for Icon protein at 5 μg/ml vs. 10 μg/ml and 20 μg/ml and P = 0.534 for 10 μg/ml vs. 20 μg/ml by unpaired t test) (Figure 2c). However, mouse Icon-mediated CDC killed only about 33% specific lysis of the cancer cells at 20 μg/ml and 25% at 10 μg/ml of Icon protein (Figure 2c), which was less than that of Icon-NK cell ADCC (Figure 2b) (70% specific lysis by NK cells with 10 μg/ml Icon and E:T ratio of 10:1, P < 0.02 by ANOVA single factor when Icon concentration was 10 μg/ml for CDC vs. ADCC). We conclude that Icon-NK ADCC mediates a stronger killing effect than Icon-CDC for TCA8113 cancer cells.

### Icon protein has no direct effect on cancer cell proliferation in the absence of NK cells and complement

**Figure 3:**
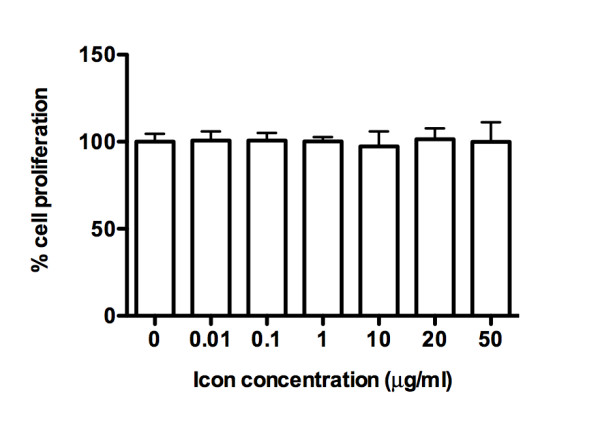
**Icon protein has no effect on cancer cell proliferation in the absence of NK cell and complement**. After incubation with mouse Icon protein for 1 hr followed by overnight growth, cancer cell proliferation was determined by CellTiter One Solution reagent, as described in Methods. P values obtained were of no significance as determined by one-way ANOVA with Tukey's Multiple Comparison Test. Data are representative of two experiments.

We showed that Icon protein at various concentrations ranging from 0.01 to 50 μg/ml, which was higher than those in the ADCC and CDC experiments, did not have any effects on the proliferation of TCA8113 cancer cells (Figure 3). Thus we conclude that Icon has no direct inhibitory effect on TF expressing cancer cell proliferation in the absence of immune cells and complement. In another word, the killing effect observed in the ADCC and CDC assays above was achieved by Icon in combination with NK (Figure 2b) or complement (Figure 2c) but not by Icon alone (Figure 3).

### NK cells are also the major players *in vivo *for Icon immunotherapy of human tongue cancer in SCID mice

To test the role of NK cells in Icon immunotherapy *in vivo*, the efficacy of Icon immunotherapy for human tongue cancer was compared between the SCID/CB-17 (NK cell functional) and SCID/Beige (NK cell defective) mouse strains.

**Figure 4:**
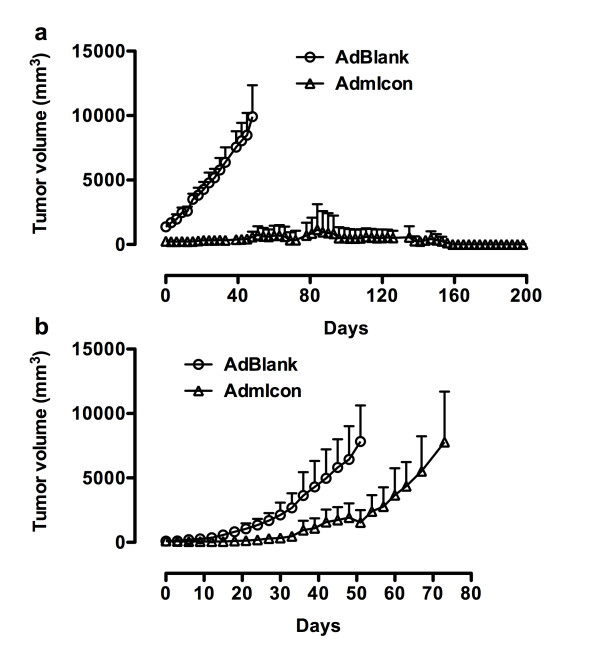
**NK cells are crucial for the efficacy of Icon immunotherapy of human tongue cancer *in vivo *in SCID mice**. Human tongue tumour xenografts were generated separately in SCID/CB-17 (a) and SCID/Beige mice (b) by subcutaneous injection of the TCA8113 tumour cells into the right flank of each mouse. After tumour formed, adenoviral vector encoding mouse Icon (AdmIcon) or control vector (AdBlank) were injected intratumourally with 1 × 10^10 ^viral particles (vp) per injection for immunotherapy on the days as indicated below. **a**: In CB-17 SCID mice, AdmIcon (◯, n = 4) or AdBlank (Δ, n = 3) was injected on days 0, 3, 6, 9, 12, 15, 18, 21. Additional intratumoural injections of AdmIcon were administered in 2 partially responded mice on days 54, 84, 93, 99 and 135. P < 0.001 on days 6 and 9 and < 0.001 on days 12-48 for AdmIcon vs. AdBlank by two-way ANOVA. **b**: In Beige SCID mice, AdmIcon (◯, n = 5) or AdBlank (Δ, n = 5) was intratumourally injected on days 0, 3, 6, 9, 12, 15, 18, 21, 24, 27, 36 and 42. The control mice were euthanised on day 53 and the Icon-treated mice on day 74. P < 0.01 on day 36 and < 0.001 on days 39-51 by two-way ANOVA for AdmIcon vs. AdBlank.

In SCID/CB-17mice (Figure 4a), TCA8113 tumours in the control group (n = 3) grew rapidly and the mice had to be euthanised on day 50 (tumour weights 8.29 ± 2.96 g on day 50, mean ± SD). In contrast, tumour volume in the Icon treated mice (n = 4) decreased to 87% by day 3 after the first injection of Icon vector and tumours were completely eradicated in 2 of 4 Icon-treated mice separately on day 60 and day 69. These mice were then tumour free for the rest of experimental period (more than 130 days) (Figure 4a). The other two Icon-treated mice also initially responded to Icon treatment and their tumours became necrotic, observed as ulcerations on the tumours on day 12, even though the tumour size was small (about 250 mm^3^). Tumour ulceration was not observed in any control tumours even when the tumours reached much larger volumes (9900 mm^3^), suggesting that tumour ulceration, which occurred in all 4 treated mice, was a sign of the therapeutic effect of Icon treatment and not due to spontaneous tumour necrosis. However, tumours in the 2 partially responsive mice still grew from the surrounding tumour margins on day 21 after the first series of injections. A second series of injection with Icon vector was completed in these two mice. Further Icon treatment caused bigger ulceration but still did not eradicate tumours. Nevertheless, Icon-treated tumour weights were 1.59 ± 1.98 g (mean ± SD) on day 156, which were significantly smaller than those of control tumours on day 50 (P = 0.015 by ANOVA single factor).

In Beige mice (Figure 4b), tumour growth in the Icon-treated mice (n = 5) was inhibited by Icon therapy as compared to the control tumours (n = 5) (P < 0.01 on day 36 and < 0.001 on days 36-51 for AdmIcon vs. AdBlank by two-way ANOVA). But by day 72, tumours reached about 7800 mm^3^, a similar size to the control tumours on day 51. The control mice had to be euthanised on day 53 and the Icon-treated mice on day 74. There was no significant difference in tumour weights between control tumours (4.99 ± 2.11 g on day 51) and Icon-treated (8.97 ± 4.06 g on day 74) tumours (P = 0.110 by ANOVA single factor). Tumour ulceration was not observed in any of control Beige mice but was observed in one of 5 Icon-treated Beige mice on day 51.

The results in these two SCID strains indicated that Icon could completely eradicate human tongue tumours in the CB17 strain that had functional NK cells, but did not achieve the same effect in the Beige strain that did not have functional NK cells, suggesting that NK cell activity is crucial and responsible for Icon to completely eradicate the established tumours. Assay for NK cell percentage in the Icon-treated mice below further indicated that not only the functional NK cells but also the NK cell percentage in peripheral blood lymphocytes (PBL) is crucial for Icon to completely eradiate tumour in host mice. Taking these *in vivo *and *in vitro *results together, we conclude that NK cell is the major player, as compared to complement, in the Icon immunotherapy for complete eradication of cancer.

All SCID mice were examined morphologically at the end of the experiments. In CB-17 mice, enlarged spleens were observed in all control mice and 2 Icon-treated PR mice. The spleens were normal in the Icon-treated CR mice. None of the CB-17 mice had metastatic tumours. In Beige mice, enlarged spleens were observed in 3 of 5 control mice and 1 of 5 of the Icon-treated mice. One control Beige mouse had one metastatic nodule (2 mm in diameter) in the lung and one Icon-treated Beige mouse had 3 metastatic nodules (2 mm in diameter) in the lungs. There were no other abnormalities observed in the SCID mice.

### NK cells can prevent tumour formation and their percentages in PBL are closely correlated with the efficacy of Icon-mediated immunotherapy in SCID mice

Using flow cytometry with an NK cell specific antibody DX5-FITC conjugate, we analysed the NK cell percentages in PBL from normal SCID/CB-17 and SCID/Beige mouse strains. In female SCID/CB-17 mice, the NK percentage in PBL was 18.2 ± 5.2% at 4-weeks old (n = 9) and 13.7 ± 5.3% at 9-10 weeks old (n = 10) with an average of 16% at age of 4-10 weeks old (*p *= 0.0775 between 4 weeks old and 9-10 weeks old). In female SCID/Beige mice, the NK percentage was 12.9 ± 1.1% at 4 weeks old (n = 3) (p = 0.116 as compared to the CB-17 strain at age of 4 weeks old), indicating that NK cells are present in peripheral blood of the Beige mouse strain. However, it is notable that NK function was depressed by Beige gene mutation [31,32].

To establish tumours for experiments in Figure 4a and 4b, 10 SCID/CB-17 mice and 10 SCID/Beige mice were separately injected s.c. with the same number (6 × 10^5 ^cells) of human tongue cancer TCA8113 cells. Within one month, all of 10 SCID/Beige mice evenly developed tumours with an average of 270 mm^3^. However, it took 2 months for 4 of the 10 CB-17/SCID mice to develop tumours with similar volume to the SCID/Beige mice. Additionally, in this strain, another 3 had tumours larger than those observed in the SCID/Beige mice (1300 mm^3^) and the last 3 CB-17/SCID mice never developed tumours. Similarly, in an unpublished experiment there were another 10 SCID/CB-17 mice, 8 of which developed skin tumours early. The tumour size also varied in these SCID/CB-17 mice. The difference in tumour growth between the CB-17 and Beige SCID strains suggest that functional NK cells in the CB-17 strain played a role in rejecting or delaying tongue cancer formation.

To understand the variations in Icon efficacy between the CR and PR SCID/CB-17 mice (Figure 4a), the NK percentages in peripheral blood lymphocytes (PBL) were monitored by flow cytometry 29 days before and at the time of euthanisation. The Icon-treated CR mouse had a normal NK cell percentage at 14.2% at 29 days before enthanisation, whereas the NK level was 14.0% in a normal SCID/CB-17 mouse of the same age. However, the NK level in the Icon-treated PR mouse was 9.6% at 29 days before euthanisation and further significantly decreased to 3.7 ± 0.9% at the time of euthanisation. At the same time, the CR mouse had 12.8 ± 2.0% of NK in PBL (*p *< 0.03 by ANOVA single factor for CR mouse vs. PR mouse), suggesting that an impaired NK level could contribute to the faster tumour growth in 3 of the CB-17/SCID mice above and to the resistance to Icon immunotherapy observed in these PR mice (Figure 4a).

## Discussion

We have previously shown that Icon immunotherapy was efficacious for selective eradication of pathological neovasculature for the treatment of several types of cancer [8-10,23], wet macular degeneration [24,25] and endometriosis [26]. But its mechanism of action was previously unclear. In this paper, we elucidate the mechanisms of action of Icon immunotherapy for cancer *in vitro *and *in vivo*. We show that (i) both ADCC and CDC are involved in mechanisms of action of Icon immunotherapy, but NK cells play a more important role than complement in mediating antibody-like Icon immunoconjugate to kill cancer cells; and (ii) the activity and level of NK cells are crucial for Icon to completely eradicate cancer in mice. We have also ruled out the possibility that Icon has any direct effects on killing or inhibiting cancer cell proliferation. We understand that the role of complement in mediating Icon immunotherapy can be further elucidated in complement-deficient mice. However, so far these complement-deficient mouse strains are derived from immunocompetent mice, for example, C3^-/-^C4^-/- ^was from C57Bl/6 background [34], in which human tumours cannot be generated. Therefore, further tests for the role of complement in Icon immunotherapy will be completed using mouse tumour cell lines in future studies.

NK cells played at least two distinct roles in this study, the first being to prevent tumour development after inoculation of tongue cancer cells by non-specific killing and the other to mediate ADCC by Icon-guided specific killing after Icon immunotherapy is initiated. The first role of NK cells was observed as the difference of tumour formation/volume between CB-17 and Beige strains. Previous reports also showed that NK cells could mediate ADCC for other therapeutic antibodies including trastuzumab (Herceptin) for breast cancer [35], rituximab (Rituxan) for B-cell lymphoma [36], and Cetuximab for lung cancer [37].

It has been observed that NK cell percentage/activity can be impaired by tumours in animals and cancer patients. In 1979, Herberman RG et al. summarised in a review article [38] that NK activity had been found to be impaired in tumour-bearing mice and patients that had large tumour loads. In later years, more reports showed that the ADCC activity of NK cells was impaired in patients with cancer, including breast [39], gastric [40], esophageal [41,42], and urologic cancers [43] as well as leukemia [44-46] and myeloma [47]. In addition, NK activity can decline with age [48], which may contribute to higher incidence of cancer developed in elderly populations. With regard to the role of NK cells in Icon immunotherapy for tongue cancer, we observed in this study that Icon immunotherapy was able to completely eradicate tumours in SCID/CB-17 mice that had functional and normal levels of NK cells but not in the Beige mice that did not have functional NK cells. Icon was also unable to completely eradicate tumours in some of the PR CB-17 mice in which NK cell percentages were lower than the normal level (average 16% of PBL in 4-10 weeks old CB-17/SCID mice).

Considering the previous observations regarding impaired NK cell activity in cancer patients and in mice, as reviewed above, with our observations in this study, we hypothesize that decreased/depressed NK cell level/activity can contribute to resistance to Icon and other antibody immunotherapies for cancer. Resistance to antibody immunotherapy has been observed in preclinical studies and clinical trials. For example, the rate of primary resistance to single agent Her2-targeted trastuzumab immunotherapy was 66% to 88% of patients with metastatic breast cancer that overexpressed the Her2 antigen [49]. Varchetta et al. analysed circulating mononuclear cells in 18 breast cancer patients after treatment of Her2 targeted-trastuzumab therapy and found that NK cells and CD56+ T cells were involved in trastuzumab treatment and concluded "quantity and lytic efficiency of CD16+ lymphocytes [i.e., NK cells] are major factors for ADCC induction by trastuzumab" [50].

If decreased NK cell level/activity is detected in host (tumour-bearing mice or patients), Icon immunotherapy should be combined with NK cell infusions and/or NK-activating cytokines. Infusions of autologous or allorgeneic NK cells for cancer therapy have been shown feasible and well-torelated in cancer patients [51-53]. We will test if infusion of NK cells could amplify the Icon efficacy in future preclinical and clinical studies. In addition, efforts are being made in our laboratory in generating immortalized NK cell lines. If successful, these NK cell lines could be used for ADCC assays *in vitro *and potentially for immunotherapy *in vivo *in combination with Icon or other therapeutic antibodies.

Previous *in vitro *and *in vivo *studies have shown that cytokines such as IL-2, IL-12, IL-15 and interferon can enhance the effect of antibody-ADCC for immunotherapy of cancer, e.g., IL-2 and IL-15 enhanced NK cell response to Herceptin-coated Her2/neu+ breast cancer cells *in vitro *[54]. Using an adenoviral vector encoding mouse IL-12 in combination with AdmIcon vector, we indeed observed that the efficacy of Icon immunotherapy was significantly enhanced by mIL-12 for the treatment of human tongue cancer in SCID/CB-17 mice and murine breast cancer in Balb/c mice (Hu et al., unpublished data).

In this regard, monitoring the NK level in PBL is important for interpreting the efficacy of Icon and other antibody immunotherapy. In this paper, we use a simple flow cytometry procedure for this purpose, in which a commercial anti-pan NK DX5 antibody FITC or PE conjugate is first incubated with a small volume (100 μl) of blood samples containing anti-coagulant such as heparin or citrate sodium followed by lysis of RBC and then cell sorting using flow cytometry. This procedure can be easily adapted for clinical trials using anti-human NK antibody fluorescent dye conjugates. We recommend that NK level/activity should be monitored and tested as a standard operation protocol in cancer patients before and during the treatment with antibody immunotherapy.

## Conclusions

In conclusion, this paper reports for the first time the efficacy and the mechanism of action of the novel, dual neovasculature- and cancer cell-targeting fVII/Fc Icon immunotherapy for human tongue cancer *in vitro *and *in vivo*. The importance of NK cell level/activity for the underlying mechanism of Icon immunotherapy could also be true in the animal models of age-related macular degeneration and endometriosis, in which Icon immunotherapy was effective in eradicating the pathological neovasculature [24-26]. In addition, our observations on the importance of NK cells can also have implications for other immunotherapeutic antibodies that are currently under investigation in preclinical and clinical studies.

## Methods

### Cell lines

Human tongue cancer TCA8113 cell is a squamous cell carcinoma line derived from human tongue cancer [28] and was kindly provided by Dr. Wei Guo at Shanghai Medical University in China. TCA8113 and Chinese Hamster Ovary (CHO) cells (ATCC) were grown in RPMI 1640 supplemented with 10% FBS and antibiotics. 293 cells (ATCC), as adenoviral packaging cells, were grown in DMEM with 10% FBS and antibiotics.

### Construction of plasmid and synthesis of Icon (fVII/IgG1 Fc) protein

The expression plasmid DNA vector pcDNA3.1(+) encoding murine fVII/hIgG1 Fc has been previously constructed and transfected into CHO cells to generate stable Icon producer cell lines [8-10]. For Icon protein production, the cloned Icon producer CHO cells were first grown in serum containing RPMI 1640 medium. When the cells reached approximately 85-90% confluency, they were switched to a serum-free Excell 301 medium (JRH Biosciences) supplemented with 1 μg/ml vitamin K1 for γ carboxyl posttranslational modification to fVII protein [55]. The SFM medium was collected twice a week and centrifuged. Affinity purification of Icon protein was accomplished using Protein A [8] with modifications as follows. After the SFM supernatant was mixed with 1/50 volume of 1 M PB pH7.0 and filtered through a 0.45 μm filter (Millipore), the supernatant was loaded by pump at less than 5 ml/min speed to a 5-ml HiTrap rProtein A column (Amersham Pharmacia) followed by wash with 20 mM PB pH 7.0 and elution with 0.1 M Glycine-HCl pH2.8. The eluate was neutralised immediately with 1/10 volume of 1 M Tris-HCl pH 9.0 and the fractions containing the Icon protein were combined and dialysed against HEPES buffer (10 mM HEPES pH7.0, 150 mM NaCl, 5 mM CalCl_2_) using a Slide-A-Dialysis Cassette (Pierce). The molecular weights of the monomer and dimer of the mfVII/hFc protein were determined by SDS-PAGE. The binding activity of purified Icon protein was tested by fluorescence-activated cell sorting (FACS) using human tongue cancer TCA8113 cells, following our earlier procedure [8-10].

### Preparation of adenoviral vectors

The procedure of constructing the adenoviral shuttle vector encoding the Icon and making the adenoviral vector particles has been described previously [8] using the AdEasy vector system with a modification to provide dialysis of the vector. The Icon vector used in this study separately encodes mouse Icon and green fluorescent protein (GFP) (AdmIcon). The control vector encodes GFP but does not encode Icon (AdBlank). Briefly, the adenoviral vectors were first amplified in 293 cells and were purified by CsCl gradient ultracentrifugation followed by dialysis using a Slide-A-Dialysis Cassette (Pierce, 3-12 ml) against 10 mM Tris-HCl pH 8.0, 2 mM MgCl_2 _and 4% sucrose, instead of PBS as previously used, then aliquoted and stored at -80°C. The reason for this modification was based on a report showing that infectious activity of the adenoviral vector could be preserved longer in Tris buffer than in PBS [56].

### Western blotting analysis of Icon in the culture medium of tumour cells after infection with Adenoviral vector encoding Icon

A similar procedure was described earlier [9]. The human tongue cancer TCA8113 cells were grown in RPMI 1640/10% FBS in a 6-well plate and then infected with AdmIcon at 50 of multiplicity of infection by incubation at 37°C for 90 min. After infection, the tumour cells were washed and then were grown in serum free medium supplemented with 1 μg/ml vitamin K1 (Sigma). The cells were observed and photographed under fluorescent microscopy. The serum free culture medium was tested for Icon protein production as follows. One ml supernatant of collected serum free culture medium was mixed with 1/50 volume of 1 M PB pH7.4 and 10 μl of Protein A Plus resin (Pierce) at 4°C overnight, and then the captured Icon protein on Protein A resin was eluted with 1 × SDS loading buffer and analysed by SDS-PAGE and Western-blotting using 0.5 μg/ml of anti-human IgG Fc HRP conjugate (Vector Laboratories) followed by ECL reagents (Pierce).

### Isolation of murine splenic NK cells for ADCC assay

Mouse NK cells were isolated from splenocytes of 4-6 weeks old female CB-17/SCID mice by EasySep Murine NK positive selection kit (Stem Cell Technologies) following the manufacturer's instructions with modification to remove red blood cells. To remove RBS, FACS lysolution (150 mM NH_4_Cl, 1 mM KHCO_3_, 0.1 mM EDTA pH 7.3) was added to the splenocyte single cell suspension with a ratio of 30:1 (FACSLysolution:Cell suspension) and incubated at RT for 30 min. After isolation, 100 μl of NK and non-NK cells were sorted by flow cytometry to assure the purity.

### Icon-mediated ADCC and CDC assays *in vitro*

Human tongue cancer TCA8113 cells were seeded into U-bottom 96-well plates (1 × 10^4 ^cells per well) and grown overnight. The following day, the cells were washed 4 times with Hanks' balanced salt solution (HBSS) containing 5 mM of CaCl_2 _and were labeled with Calcein-AM (Molecular Probe/Invitrogen) by incubation to a final concentration of 8 μM Calcein-AM in HBSS at 37°C for 40 min. After one wash, the cells were incubated quadruplicate with mouse Icon protein or HBSS buffer only at 37°C for 20 min. and the plates were used for CDC or ADCC as follows.

For CDC assay, we used rabbit complement but not human complement because it was reported that rabbit serum had stronger complement activity than that from several other species including humans [57]. 1:4 diluted rabbit complement MA (Cedarlane Laboratories) in HBSS was added to each well that had been incubated with mouse Icon protein (Icon-CDC experimental wells) or HBSS buffer (complement alone wells) and incubated at 37°C for 60 min. For the ADCC assay, freshly isolated mouse NK and non-NK cells were added to the plates and incubated at 37°C for 4 hrs. In the NK cell alone control wells, only the HBSS buffer was added without Icon (in order to determine the direct effect of NK cells). For both assays, only HBSS buffer was added without Icon in the spontaneous release wells. In the maximal release wells, 1/10 vol (10 μl) of 9% Triton-X 100 was added to HBSS to lyse the cells. Supernatant from each well was transferred to a new 96-well plate and fluorescence was measured on a fluorescence microplate reader (Bio-Tek) with excitation 485/20 nm and emission 528/20 nm. The percent specific lysis of cancer cells by Icon-CDC was calculated using the following formula: (Fluorescence in the experimental well-Mean Fluorescence in the complement alone wells)/(Mean Fluorescence in the maximal release wells-Mean Fluorescence in the complement alone wells) × 100%. Percent specific lysis of cancer cells by Icon-NK ADCC was calculated with the following formula: (Fluorescence in the Icon/NK cell well-Mean Fluorescence in the NK cell alone wells)/(Mean Fluorescence in the maximal release wells-Mean Fluorescence in the NK cell alone wells) × 100%.

### Cancer cell proliferation assay

Human tongue cancer TCA8113 cells were seeded at a concentration of 2 × 10^4 ^cells per well in a 96 well plate and grown in complete growth medium supplemented with 10% heat inactivated FBS overnight. Then the cancer cells were incubated with mouse Icon protein diluted in HBSS/1%BSA/10 mM CaCl_2 _at concentrations of 0, 0.01, 0.1, 1, 10, 20, or 50 μg/ml in quadruplicate at 37°C for 1 hr. After removing the Icon solution, 100 μl of complete growth medium was added to each well and the cells were grown at 37°C and 5% CO_2 _overnight. 20 μl of CellTiter One Solution (Promega) was added to each experimental well and medium background control wells and incubated at 37°C and 5% CO_2_. The medium background control contained growth medium without cells. OD490 nm was measured separately at 4 hrs. The cell proliferation percentage was calculated as (experimental well-growth medium background control)/(no treatment control-growth medium background control) × 100%.

### Tumour xenograft models in mice

The protocol for the animal studies was approved by the Yale University Institutional Animal Care & Use Committee. Human tongue tumour xenografts were separately generated in 4-6 weeks old female SCID/CB-17 and SCID/Beige mice (Taconic Farms Inc.) by subcutaneous (s.c.) injection of 6 × 10^5 ^cells in 100 μl PBS.

### Icon-mediated Immunotherapy for treatment of human tongue tumours in SCID mice

After human tongue tumours formed under the skin of SCID mice, the mice were injected intratumourally with 1 × 10^10 ^viral particles per injection of an adenoviral vector encoding mouse Icon (AdmIcon), or a control vector, AdBlank, which did not encode any therapeutic agent as a blank vector control. The tumour volume (mm^3^) was calculated by measuring two dimensions in millimeters (mm) and using the formula (width)^2 ^× length/2.

### Flow cytometric analyses of NK cell percentage in mouse peripheral blood lymphocytes

The NK cell percentage in peripheral blood lymphocytes was determined by flow cytometry using anti-mouse NK DX5-FITC conjugate (CALTAG). 100 to 200 μl of a blood sample was taken by alternatively retro-orbital blood sampling in mice with heparinised blood collecting tubes (Chase Scientific) and mixed in tubes containing heparin (Sigma). Two μl of DX5 FITC conjugate was added to each 100 μl of heparinised blood in a 15-ml tube and incubated on ice for 30 min. Red blood cells were then lysed by adding 3 ml of FACS lysolution into each tube and incubating at room temperature for 10 min. The leukocytes were spun down by centrifugation at 300 g for 3 min and were washed twice with 3 ml of FACS buffer (PBS pH7.4, 1% BSA, 0.05% NaN_3_) and centrifuged at 300 g for 3 min. After the final wash, cells were resuspended in 300 μl of PBS/2 μg/ml propidium iodine (PBS/PI). The cells were sorted with BD FACS machine. The percentage of fluorescent peak shift was measured based on a control in which the DX5 FITC conjugate was not added and the result was presented as the NK cell percentage in peripheral blood lymphocytes.

### Statistical analysis

The data concerning the *in vitro *and *in vivo *effects of Icon were presented as mean values ± standard deviation and were analysed for significance between the treated groups and the control groups by Prism 5 version 5.0c (GraphPad Software Inc) and/or Microsoft Excel using paired or unpaired t tests and one-way ANOVA with Tukey's Multiple Comparison Test or two-way ANOVA methods. *P *values less than 0.05 were considered statistically significant. For analyses of statistical significance, quadruplicate wells in each group were used for ADCC and CDC assays *in vitro *in tissue culture plates and 5 mice per group were used in *in vivo *animal studies unless specified.

## Competing interests

ZH is the co-inventor of the U.S. patent on Neovascular-Targeted Immunoconjugates (U.S. Patent no. 6,924,359).

## Authors' contributions

ZH designed and performed the research, analysed the data and wrote the paper. JL performed the research and analysed the data. Both authors read and approved the final manuscript.
